# Incretin secretion stimulated by ursodeoxycholic acid in healthy subjects

**DOI:** 10.1186/2193-1801-2-20

**Published:** 2013-01-22

**Authors:** Masanori Murakami, Naoko Une, Maiko Nishizawa, Sayaka Suzuki, Hideki Ito, Toshiyuki Horiuchi

**Affiliations:** 1 Division on Endocrinology and Metabolism, Tokyo Metropolitan Health Medical Treatment Corporation Toshima Hospital, Itabashi, Japan; 2 Tokyo Metropolitan Geriatric Hospital, Itabashi, Japan

**Keywords:** Bile acid, Ursodeoxycholic acid, Glucagon-like peptide-1

## Abstract

Bile acids play an important role in post-prandial glucose metabolism by stimulating release of glucagon-like peptide-1 (GLP-1) via the G-protein-coupled receptor TGR5, which is expressed in intestinal L cells. Thus, bile acid sequestrants are expected to stimulate secretion of endogenous GLP-1 through TGR5. We investigated incretin and insulin secretion after a meal with and without ursodeoxycholic acid (UDCA), a widely used therapeutic agent in liver diseases, in 7 non-diabetic Japanese subjects. We found that UDCA intake resulted in higher GLP-1 secretion (area under the curve [AUC] of 0–60 min after meal without UDCA, 450 ± 162 mmol·min/l; with UDCA, 649 ± 232 mmol·min/l, *P* = 0.046) and lower blood glucose (AUC of 0–60 min without UDCA, 7191 ± 250 mg·min/dl; with UDCA, 6716 ± 189 mg·min/dl, *P* = 0.001) , although we did not find statistically significant insulin increase by UDCA intake (AUC of 0–60 min without UDCA, 1551 ± 418 μU·min/ml; with UDCA, 1941 ± 246 μU·min/ml, *P* = 0.065). These results suggest that UDCA increases bile-induced GLP-1 secretion. Ours is the first report showing increased GLP-1 secretion and decreased blood glucose in response to UDCA.

## Background

Glucagon-like peptide-1 (GLP-1) is major incretins released from gut endocrine cells in response to nutrient ingestion. Notably, GLP-1 not only stimulates insulin biosynthesis and secretion and promotes beta cell proliferation, but also inhibits glucose production and glucagon secretion by the liver, and reduces appetite and food intake (Nauck et al. 1989; Kreymann et al. 1987; Trumper et al. 2001; Xu et al. 2006; Prigeon et al. 2003; Flint et al. 1998; Gutzwiller et al. 1999). For these reasons, drugs that increase GLP-1 activity have become attractive therapeutic options for patients with type 2 diabetes (Campbell and Miller 2009). GLP-1 receptor agonists (incretin or GLP-1 mimetics) and dipeptidyl peptidase-4 (DPP-4) inhibitors (CD26 antigen inhibitors) are the currently available incretin-based therapies proven to be safe and effective in the management of type 2 diabetes (Amori et al. 2007). However, they do not increase secretion of endogenous GLP-1, and therapies that directly target intestinal L cells to stimulate secretion of GLP-1 are expected to be effective.

Bile acids, the principal constituents of bile, along with cholesterol, phospholipids, and bilirubin, have been shown to stimulate GLP-1 secretion. GLP-1 levels were found to increase in the portal effluent, following the luminal perfusion of bile into perfused rat colon preparations (Plaisancie et al. 1995). Furthermore, bile acid sequestrants have been found to increase plasma GLP-1 levels in human and rodents (Chen et al. 2010; Shang et al. 2010; Garg et al. 2011; Suzuki et al. 2007). In addition, high concentrations of bile acids in direct contact with L-cell-rich regions of the intestine may contribute to the rapid remission of type 2 diabetes after gastric bypass surgery (Nakatani et al. 2009; Patti et al. 2009). Recently, a bile acid-sensitive G-protein-coupled receptor (GPCR), TGR5, was described (Maruyama et al. 2002), whose activation results in enhanced GLP-1 secretion (Katsuma et al. 2005; Thomas et al. 2009). In this study, we investigated whether ursodeoxycholic acid (UDCA), a widely used bile acid sequestrant used therapeutically in liver diseases, induces GLP-1 secretion, and assessed its effects on glucose and insulin levels in healthy subjects.

## Results

In this study, we examined 7 subjects (4 men and 3 women). The mean age of the patients was 33.4 ± 7.8 years, and BMI was 20.4 ± 2.4 kg/m^2^ . All the subjects were checked in the periodical health examination and checked for normal.

**Figure 1:**
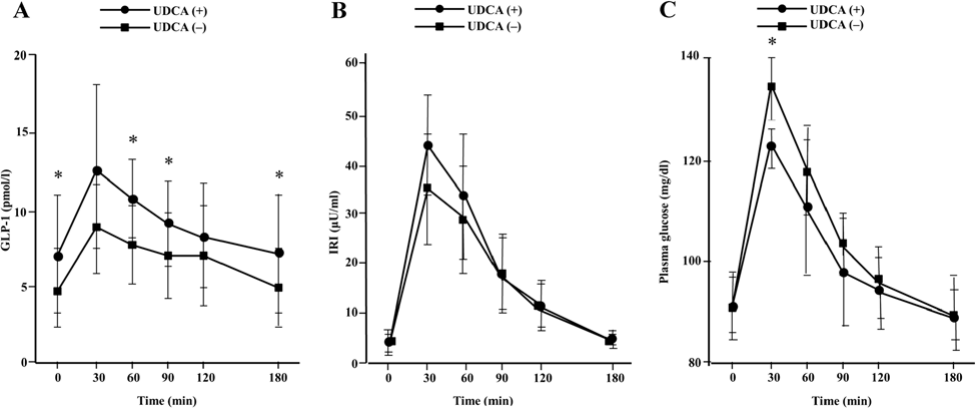
**Results of meal test for (A) plasma glucose, (B) immunoreactive insulin, (C) active GLP-1.** Data are means ± SD. Comparisons were performed using paired Student’s t-test or Wilcoxon signed rank test. * *P* < 0.05, without UDCA vs. with UDCA.

**Figure 2:**
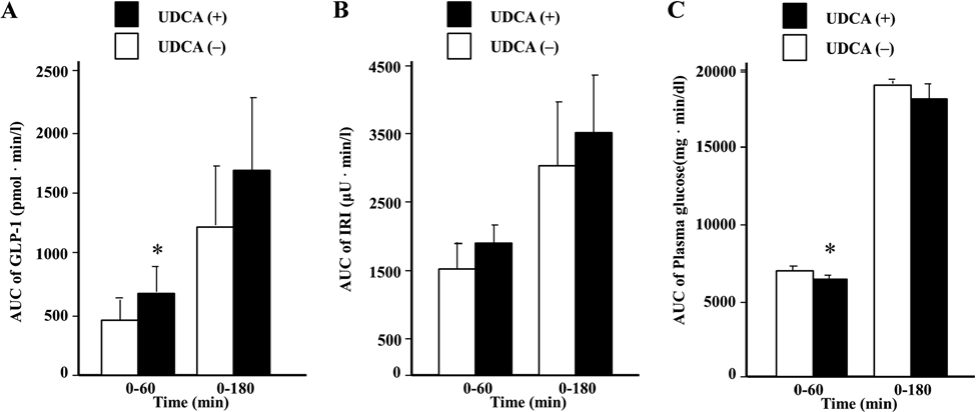
**Results of meal test for AUC of (A) plasma glucose, (B) immunoreactive insulin, (C) active GLP-1.** Data are means ± SD. Comparisons were performed using paired Student’s t-test or Wilcoxon signed rank test. * *P* < 0.05, without UDCA vs. with UDCA.

Mean changes in plasma glucose, serum IRI, and plasma active GLP-1 levels, with and without UDCA, are shown in Figure 1. A significant decrease in plasma glucose levels, comparing to UDCA intake, was observed at 30 min (without UDCA, 135 ± 6 mg/dl; with UDCA, 123 ± 3.0 mg/dl, *P* = 0.012, Figure 1A). Serum IRI levels tended to increase after UDCA intake, but we found no statistically significant change at any time point (Figure 1B). However, plasma active GLP-1 levels were increased by UDCA intake. Significant increases were found at 60, 90, and 180 min (Figure 1 C). In addition, we examined AUCs to determine whether UDCA has a significant impact on metabolic parameters (Figure 2). We found a significant decrease in AUC of 0–60 min for plasma glucose (without UDCA, 7191 ± 250 mg·min/dl; with UDCA, 6716 ± 189 mg·min/dl, *P* = 0.001; Figure 2A), but no significant change over 0–180 min (without UDCA, 19,074 ± 273 mg·min/dl; with UDCA, 18,223 ± 842 mg·min/dl, *P* = 0.050; Figure 2A). With respect to serum IRI, we found no significant increase in AUCs over 0–60 and 0–180 min (without UDCA, 1551 ± 418 μU·min/ml; with UDCA, 1941 ± 246 μU·min/ml, *P* = 0.065; without UDCA, 3117 ± 912 μU·min/ml; with UDCA, 3590 ± 794 μU·min/ml, *P* = 0.165, respectively; Figure 2B). Finally, we found a significant increase in plasma active GLP-1 levels at AUC of 0–60 min (without UDCA, 450 ± 162 mmol·min/l; with UDCA, 649 ± 232 mmol·min/l, *P* = 0.046; Figure 2C); however, over 0–180 min, we found no significant change (without UDCA, 1230 ± 485 mmol·min/l; with UDCA, 1664 ± 588 mmol·min/l, *P* = 0.060; Figure 2C).

## Discussion

To our knowledge, our study is the first to reveal a significant increase in postprandial plasma GLP-1 levels accompanied by a significant decrease in postprandial plasma glucose levels by UDCA intake in healthy subjects. We propose that these effects, increased GLP-1 and decreased glucose, explain, at least in part, the blood glucose-lowering action of UDCA, through increased plasma active GLP-1 levels resulting in higher serum insulin levels. Our data and recent findings on TGR5, which is a bile acid-sensitive GPCR (Maruyama et al. 2002) that enhances GLP-1 secretion (Katsuma et al. 2005; Thomas et al. 2009), suggest that UDCA stimulates GLP-1 secretion via TGR5 signaling. Furthermore, it was reported that a low carbohydrate/high-fat diet stimulates postprandial GLP-1 levels in healthy subjects (Numao et al. 2012). Because a high-fat diet elevates the fecal concentration of bile acids (Cummings et al. 1978; Bianchini et al. 1989), it is likely that bile acid induced by high-fat diet stimulates GLP-1 secretion. It follows that incretin hormones may stimulate insulin secretion through bile acids induced by fat diet, in addition to the widely known glucose-dependent pathway (Nauck et al. 1986).

In our study group, we observed a rapid transition of plasma glucose, serum IRI, and plasma active GLP-1 levels. The AUCs, which showed significant differences only over 0–60 min for plasma glucose and GLP-1, suggest that UDCA works transiently in an early period after administration.

Incretin-based therapies such as GLP-1 receptor agonists and DPP-4 inhibitors are currently in use for the management of type 2 diabetes. However, these patients may retain GLP-1 secretion ability, which should be considered when planning treatment. UDCA is a candidate factor for evaluating GLP-1 secretion ability, because consumption of this drug increases plasma active GLP-1 secretion in healthy subjects, as indicated by our results. We evaluated the effect of UDCA only in healthy subjects; further study in type 2 diabetes patients is necessary to test this idea. Furthermore, UDCA is usually administered to patients with liver cirrhosis or chronic hepatitis, frequently suffering from abnormal glucose metabolism. It is possible that UDCA improves glucose intolerance in those patients. For example, nonalcoholic steatohepatitis patients treated with high-dose UDCA experienced significant reductions in serum glucose, glycosylated hemoglobin, and serum insulin levels (Ratziu et al. 2011).

Interestingly, there was no statistically significant difference in insulin secretion (Figures 1B and 2B) induced by UDCA. A previous study showed that a high-fat diet, which induces bile acids secretion, stimulates GLP-1 secretion and lowers insulin levels transiently in the early phase (Numao et al. 2012). Thus, the effect of UDCA on insulin secretion should be evaluated on a finer time scale.

One limitation of our study is the small sample size. We studied only 7 subjects and the results varied widely. For example, statistically significant differences in plasma active GLP-1 levels were found unexpectedly at 0 min (without UDCA, 4.6 ± 2.8 pmol/l; with UDCA, 7.1 ± 3.9 pmol/l, *P* = 0.032, Figure 1C). Further study with a larger number of subjects is warranted.

## Conclusions

In conclusion, UDCA increased GLP-1 secretion in healthy subjects. We propose that this drug may be useful as a tool for evaluating incretin secretion in type 2 diabetes mellitus.

## Methods

### Subjects

Seven people were recruited for this study. All subjects were non-obese (body mass index (BMI) < 25). Written informed consent was obtained from all participants with the approval of the ethics committee of Toshima Hospital.

### Meal tolerance tests

Meal tolerance tests were performed after an overnight fast. The test meal was authorized by the Japan Diabetes Society; total caloric content of the meal was 460 kcal (carbohydrates, 56.5 g; protein, 18 g; fat, 18 g; JANEF E460F18, Q.P. Corporation, Tokyo, Japan). The test meal was ingested within 15 min. Blood samples were collected at 0, 30, 60, 90, 120, and 180 min after the meal. One week later, testing was performed again, with intake of UDCA 200 mg (5% granule, Urso, Mitsubishi Tokyo Pharmaceuticals) after the test meal.

Levels of glucose, immunoreactive insulin (IRI), and active GLP-1 in sera or plasma were measured at each time point. Plasma glucose concentrations were measured by the glucose oxidase method. IRI levels were measured with enzyme immunoassay kits. Plasma active GLP-1 samples were analyzed using ELISA (EGLP-35 K; Linco Research Inc., MO, USA). Plasma active GLP-1 concentrations were measured in plasma after addition of DPP-4 inhibitor (10 μl/ml blood; Linco Research Inc., MO, USA).

### Statistical analysis

Data are presented as means ± standard deviation (SD). Statistical calculations including areas under the curve (AUCs) were carried out using paired Student’s t-test or Wilcoxon signed rank test, as appropriate. A *P* value < 0.05 was taken to indicate a significant difference. Normality of the data was evaluated by the Shapiro-Wilk test.
